# Rheological and Biological Properties of Adhesive Skin Secretions from *Eupsophus vertebralis* (Anura: Alsodidae)

**DOI:** 10.1155/2024/2722351

**Published:** 2024-03-25

**Authors:** Felipe A. Contreras, Daniela P. Sepúlveda, André Capaldo Amaral, José J. Nuñez, Eliane Trovatti, Elkin Y. Suárez-Villota

**Affiliations:** ^1^Instituto de Ciencias Naturales, Facultad de Medicina Veterinaria y Agronomía, Universidad de las Américas, Av. Jorge Alessandri No. 1160, Concepción, Chile; ^2^Program of Post-Graduation in Regenerative Medicine and Medicinal Chemistry, University of Araraquara–UNIARA, Rua Carlos Gomes, Araraquara 1217, Brazil; ^3^Instituto de Ciencias Marinas y Limnológicas, Facultad de Ciencias, Universidad Austral de Chile, Casilla 567, Valdivia, Chile

## Abstract

Skin secretions from Patagonian ground frogs, *Eupsophus vertebralis*, have previously been reported as a potent proteinaceous adhesive with potential biomedical applications. Here, we conducted a rheological analysis indicating the mechanical robustness of these secretions, with a storage modulus ranging from 1 to 10 Pa. In addition, antimicrobial and cytotoxicity assays were performed, revealing no antimicrobial activity against both the Gram-positive and Gram-negative bacteria. The cytotoxicity results were intriguing, as three samples showed no harm, and one exhibited a severe cytotoxic effect on the human cell line MG63. These properties, as indicated by these preliminary results, reinforce their potential for practical applications in the industrial and medical sectors.

## 1. Introduction

Extensive global research into floral, fungal, and faunal biodiversity continuously seeks natural products with potential applications in human consumption, medical aid, and pharmaceutical development [1–4]. Other applications include herbicides for agricultural practices, pest control, and accelerators or retardants of biotechnological or biochemical reactions [1, 5, 6]. Among these natural products, animal extracts possess unmatched properties such as extensive biocompatibility, high adsorption capacity, and excellent film-forming substances [7]. Industrially produced animal-derived products have a wide range of applications, including controlled drug delivery systems, leather finishing, and pollutant absorption [7].

Most chemical bioproducts are discovered through interactions between organisms and their environment, providing a diverse array of complex chemical entities [8]. For example, epibatidine, derived from the skin of an Ecuadorian poison frog, is an anesthetic used by indigenous tribes in darts for hunting, and it is ten times more effective than morphine [9, 10]. While much research on amphibian skin secretions has focused on biologically active compounds responsible for their toxic, pharmacological, antimicrobial, poisonous, and therapeutic properties [2, 11], there has been comparatively less research on adhesive secretions. These amphibian secretions can fasten the breeding pair during amplexus [12]or affix dry leaves or debris to a predator's mouth, serving as a distraction and enabling the prey to escape [13]. Such secretions can complement the amphibians' defense strategy when they are distasteful or toxic [14]. Adhesive secretions have been identified in several animal specimens globally, such as those from *Mytilus edulis* and *Phragmatopoma californica* studied for biomedical uses [15]. However, the adhesive secretions from frogs have been identified in a few species, including representatives from all three amphibian orders [13], among which some can be highlighted, such as the South African frogs from the *Breviceps* genus [16, 17], *Notaden* species, a genus of Australian fossorial frogs from the *Limnodynastinae* subfamily [17, 18], and the ground frog *Eupsophus vertebralis* [19], a species distributed in Patagonia (Chile and Argentina) [20].

When threatened by potential predators, the *E. vertebralis* frog naturally secretes a sticky substance from its dorsal skin, which quickly solidifies into an elastic material that adheres firmly to a variety of surfaces, including glass, plastic, and metal, and to biological materials such as skin, bone, and cartilage [19]. The bonding strength of the *E. vertebralis* glue is among the highest in the animal kingdom and is comparable to industrial superglues such as cyanoacrylates (3.34 MPa) [19]. Dry glue from *E. vertebralis* consists of approximately 50% protein content (Bradford assay), spanning from 25 to 250 kDa [19]. In this study, we performed a rheological study to assess the mechanical robustness of the *E. vertebralis* skin secretion, antimicrobial assays using Gram-positive and Gram-negative bacteria, and also tested its cytotoxic activity against a human cell line. The *in vitro* cytotoxicity assays and the cell adhesion tests were conducted hereat the first level of evidence for biomedical applications of these secretions. We anticipate that analyzing these properties will enable us to evaluate their practical applications in industrial and medical sectors, leveraging their natural origin and biotechnological potential.

## 2. Materials and Methods

### 2.1. Sample Collection

We captured five *E. vertebralis* specimens from Punucapa, Chile (−39.767222; −73.261666) and three from Morrompulli, Chile (−39.960119, −73.124569) under the supervision and approval of the Bioethics and Biosecurity Committee of the Universidad Austral de Chile (UACh, Resolution Nos. 236/2015 and 61/15) and the Servicio Agrícola y Ganadero, Chile (SAG, Resolution No. 9244/2015). From these specimens, four of them exhibited stick secretions, which were collected using a sterile spatula directly from the skin and stored in ethanol 96% at −20°C. Subsequently, the animals were released at the same collection site. Samples were labelled as EVPN1040 and EVPN1370 from Punucapa and EVMO1417 and EVMO1418 from Morrompulli.

### 2.2. Structural and Physicochemical Characterization

#### 2.2.1. Solubility Procedure

The process began by homogenizing the samples in a 4% NaCl solution for 30 minutes. Following this, they underwent two washes with 0.5 mL of distilled water to remove the NaCl. Subsequently, the samples were dissolved in 5% acetic acid until they reached a final concentration of 0.45%, after which they were vortexed and left to rest for 24 hours. They were then dried in an oven at 40°C until completely dry, which typically took about 24 hours. Afterward, distilled water was added until the concentration reached 0.5%, and the samples were vortexed again. To prevent losses, the initial tube was retained throughout the process.

#### 2.2.2. Fourier Transform Infrared (FTIR)

The dried samples were analyzed using a Perkin–Elmer Spectrum 100 Fourier transform infrared (FTIR) spectrometer equipped with an attenuated total reflectance (ATR) device with a diamond-coated zinc selenide crystal. The spectra were recorded in the range of 650–4000 cm^−1^ with a resolution of 4 cm^−1^ and 16 scans. The spectral outputs were recorded in transmittance.

#### 2.2.3. Rheology

The rheological properties of the samples were analyzed using an Anton Paar MCR92 rheometer fitted with a 50 mm parallel plate geometry to evaluate the viscoelastic properties of the secretion. The sample solutions were loaded onto the rheometer plate accessory. The gap size was 0.10 mm. The test was performed at 25°C, and the viscoelastic properties, namely, the storage modulus *G*′ and the shear modulus *G*^″^, were measured in the range of 0.01 Pa and 50 Pa, with a frequency of 1 Hz in oscillatory stress sweep mode.

### 2.3. Biological Characterization

#### 2.3.1. Antibacterial Activity

The antimicrobial effect of the secretion was performed following Lu et al.'s [21] protocol, which was only carried out with samples from the Punucapa locality due to sample availability. Standard bacterial strains used in antimicrobial assays were the Gram-positive bacterium *Staphylococcus aureus* (ATCC 25923) and the Gram-negative bacterium *Escherichia coli* (ATCC 25922). Bacteria were first grown in LB (Luria–Bertani) broth to an OD600 nm of 0.8. An aliquot (10 µL) of the bacterial suspension was added to 8 mL of the fresh LB broth with 0.7% agar and poured over a 90 mm Petri dish containing 25 mL of 1.5% agar in LB broth. After the top agar hardened, a 20 *μ*L aliquot of the test sample filtered on a 0.22 *μ*m millipore filter was dropped onto the surface of the top agar and completely dried before being incubated overnight at 37°C. If a secretion displayed antimicrobial activity, a clear zone would form on the surface of the top agar, indicating the inhibition of the bacterial growth. Antibiotic-antimycotic 1X (Gibco™) was used as the positive control, and acetic acid (5%) was used as the negative control.

#### 2.3.2. Cytotoxicity Tests

Cytotoxicity tests were carried out following the standard resazurin reduction method [22]. The human MG63 cell line was used in the experiments. Two rounds of experiments were performed, using two different methods, to understand the cytotoxic effect of the samples on the cells.

The first round of the method was performed using indirect tests, with the extract of the samples, following the ISO 10993-5 standard. For the preparation of the extracts, each sample was kept in contact with Dulbecco's Modified Eagle Medium (DMEM)'s culture medium supplemented with 10% volume of fetal bovine serum (FBS) to a final concentration of 1 mg/mL, for 24 h. For the test, the cells were seeded into the wells of a 96-well microplate (1.0 × 10^4^ cells/well) in 100 uL of DMEM culture medium supplemented with 10% volume of FBS and maintained in a humidified atmosphere of 5% CO_2_ and 95% air, at 37°C, for adhesion. After 24 h, the culture medium was replaced by the sample extracts (100 *μ*L) and incubated at the described growth conditions for a further 24 h. Subsequently, the culture medium was replaced by 50 *μ*L of 0.01% wt resazurin aqueous solution. The plates were incubated for 4 hours at the growth conditions. The fluorescence was measured using a BioTek (Synergy H1) spectrofluorometer, at *λ*_ex_ = 530 nm and *λ*_em_ = 590 nm. Cell viability was determined by comparing the viability of the test samples with that of the negative control. The negative control represents 100% cell viability, and the samples' viability was calculated relative to this control. The tests were carried out in triplicate.

The second round of experiments was performed using the direct test, in which the cells were in direct contact with the extract to confirm the results of the first round and to test the cell adhesion to the surface of the hydrogels. For this, the samples from the first round of experiments were dissolved in acetic acid (5% volume) and used to coat the bottom of the wells of a 96-well microplate. After drying, the sample/microplate was sterilized under UV light for 30 minutes. The cells (1.0 × 10^4^ cells/well) were then seeded on the surface of the samples by using 100 *μ*L of DMEM culture medium supplemented with 10% volume of FBS. The microplate was maintained in a humidified atmosphere of 5% CO_2_ and 95% air at 37°C for 24 h. The cell viability was determined as described in the first experiment. The cell adhesion was visualized by fluorescence microscopy using 4′,6-diamidino-2-phenylindole (DAPI) as the fluorescent marker of cell nuclei. The tests were carried out in triplicate.

## 3. Results and Discussion

**Figure 1:**
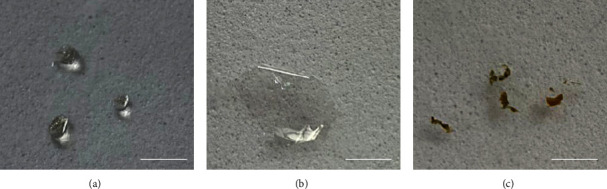
Skin secretions from *Eupsophus vertebralis* after performing the solubility protocol (see text for details): (a) viscous mucus visual aspect of the secretion before drying, (b) film formed from the dried secretion, and (c) secretion grains formed after drying. Scale bar = 500 *μ*m.

In this study, we present a comprehensive characterization of *E. vertebralis* secretions, employing various analytical techniques such as Fourier transform infrared spectroscopy (FTIR) and rheological studies, as well as assessments of antimicrobial activity and cytotoxic effects. The study of amphibian secretions has revealed significant potential for biotechnological applications, as outlined in the Introduction section [17, 18]. However, to advance this initial exploration, further investigation requires the development of protocols for solubilizing various secretions, understanding their stability under diverse conditions, evaluating their cytotoxicity, and exploring potential biomedical applications. We propose a protocol for solubilizing these (Figure 1(a)) secretions from their native hydrogel form and resolubilizing them postdrying. While protocols exist in the literature for proteinaceous secretions [17, 22], we found that some were ineffective for *E. vertebralis*. Notably, one of the secretions (EVPN1040) exhibited a filmogenic property after the drying process, resulting in a completely transparent and homogeneous film (Figure 1(b)). However, the other samples formed hydrogels or grains when dry (Figure 1(c)).

The secretions behave as a rigid polymeric glass when dried, and rubbery at higher hydration levels, similar to the behavior of elastomeric biomedical material [23]. The variability in solubility could be attributed to the nature of the “bulk discharge” observed in *E. vertebralis* secretion [19]. It is known that this type of discharge involves serous or serous-derived specialized glands that release several materials containing nuclei and components of the rough endoplasmic reticulum, along with integral secretory granules [24, 25]. The “bulk discharge” phenomenon was not observed in all *E. vertebralis* individuals, as observed in this study and previous reports [19]. In anurans, the contraction of myoepithelium, which is involved in this discharge, is regulated by an adrenergic mechanism [26]. Hence, the varying degrees of secretion release among *Eupsophus* specimens represent graded defense responses and might be influenced by the perceived level of noxious manipulation [19].

**Figure 2:**
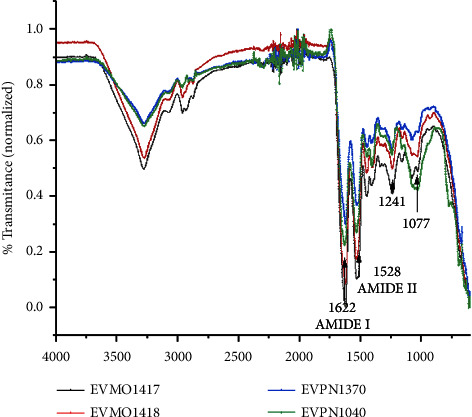
Fourier transform infrared (FTIR) spectroscopy of *Eupsophus vertebralis* secretions. Bands of amides I and II are shown. Voucher numbers of each specimen are indicated.

The FTIR analysis of the crude dried samples indicated the typical protein structure as the major contribution to the spectra of all the samples. In fact, the FTIR spectrum showed typical bands of the amide bond, with bands of about 1623–1650, 1528, and 1241 cm^−1^, corresponding to amide I, II, and III, respectively (Figure 2). Thus, all the spectra were quite similar and displayed bands of secondary structure with domains folded in *α*-helix and *β*-sheet conformation predominantly, which is in agreement with the literature [27]. Regarding the structure, amide I is associated with the C=O stretching vibration, amide II results from the N–H bending vibration and C–N stretching vibration, and amide III is a complex band resulting from several coordinate displacements [28]. Amide I vibration is strictly related to the backbone's secondary structure. Literature shows that it is possible to distinguish the secondary structure of peptides and proteins by the shape and absorption wavenumber of the amide I band [29]. The *α*-helix and random coil secondary structures show large bands at around 1642–1660 ^−1^and 1662–1686 cm^−1^ [28]. *β*-sheet conformation shows two typical peaks at around 1615–1638 and 1672–1694 cm^−1^, and if the peptide is predominant *β*-sheet, the peaks are very well separated [29]. In summary, the FTIR analysis indicated that the predominant constituent of the analyzed content comprises typical proteins. The physicochemical properties of the samples, particularly their filmogenic nature, suggest that their major component has a high molecular weight; otherwise, it would not be able to form a film.

The highly organized structure of the proteins influences the mechanical behavior of the material. Here, the secretions showed the interesting property of behaving as hydrogels. Hydrogels are crosslinked polymers which keep large amounts of water entrapped within their structure [30]. This property has been extensively studied for application in drug release and regenerative medicine for cell delivery, suggesting the potential of these biomaterials for application in the biomedical field [31].

**Figure 3:**
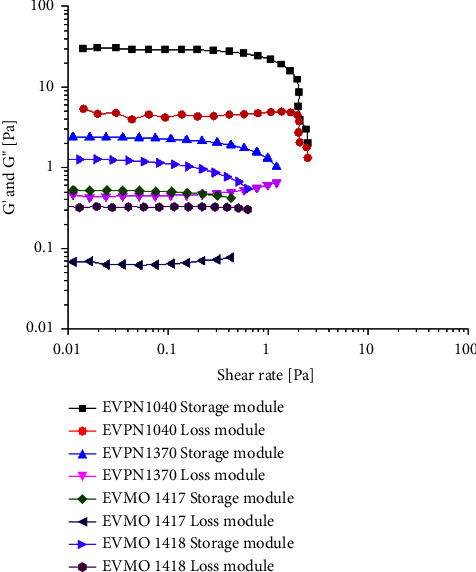
Rheological test of the *Eupsophus vertebralis* secretions. Stored deformation energy (storage module) and loss deformation energy lost (dissipated) through internal friction when flowing (loss module) are indicated for each specimen.

The linear viscoelastic region of the hydrogels was analyzed by oscillatory rheology. The results showed that the storage moduli (*G*′) of the hydrogels displayed a plateau in a frequency range of 0.1–100 rad·s^−1^, as shown in Figure 3. For low strain values, the storage modulus (*G*′) and the loss modulus (*G*^″^) were independent of the shear stress, and *G*′ > *G*^″^ was up to about 1 Pa for all *E. vertebralis* samples, indicating that the material is highly structured and mechanically robust. The high *G*′ values for the sample EVPN1040, ∼20 Pa, indicated its stronger structure under shear, when compared to the other samples, EVPN1370 (∼2 Pa), EVMO1417 (∼0.5 Pa), and EVMO1418 (∼1.5 Pa), indicating their structural deformation and the transition from elastic to viscous behavior (*G*^″^ increases). The increase of the shear rate above the critical value leads to the disruption of the hydrogel structure network, from a solid-like state to a liquid-like state. In this condition, the gel structure starts to be destroyed and gradually behaves as a fluid-like material.

The elastomeric property is also important for the development of structures for a range of biomedical applications, mainly for those used in regenerative medicine, once many tissues in the body have elastomeric character and must be resilient upon stretching [32]. Proteins can undergo deformation without rupture, because of the elastic behavior, which depends on their structure. They can efficiently store the energy of deformation and then return to its original state on unloading the deformation or stress [32]. This behavior can be explained by the protein structure, which is conformationally flexible, forming a network crosslink, to respond to external forces. These crosslink networks distribute the stress throughout the system, preventing the polymer chains' separation when the force is applied, thus avoiding destroying the structure. In the wet state, the liquid acts as a plasticizer favoring the motion of hydrogen bonds among the amino acid groups.This mantains the structure of the hydrogel and its elastic properties. In the dry state, the high force of the intermolecular interactions leads the proteins to behave like a solid because of the decrease in the segmental motions [32].

The secretion samples analyzed did not exhibit antimicrobial effects against *Staphylococcus aureus* (ATCC25923) and *Escherichia coli* (ATCC25922) (Supplementary Figure 1). As these types of secretions are a mixture of various substances with diverse concentrations (18, 19, and 27), we cannot rule out the possibility that some substances present in low concentrations may have antimicrobial effects. Therefore, additional analyses evaluating the antimicrobial effect of purified and concentrated molecules from the crude secretion will be necessary to confirm or rule out such an effect. Indeed, peptides with antimicrobial effects isolated from amphibian secretions act by destroying cell membranes, a process that requires high concentrations [33].

**Figure 4:**
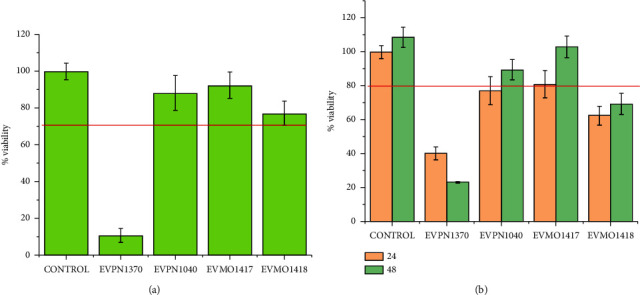
Cytotoxicity assays for *Eupsophus vertebralis* secretions using the MG63 cell line: (a) cell viability of indirect cytotoxicity tests using an extract of the secretion, following ISO 10993-5 standard, and (b) cell viability of direct cytotoxicity tests, where cells were in direct contact with the secretion (see text for details). Cell viability is shown at 24 h for direct tests and 24 and 48 h for indirect tests. The red line indicates 70% viability.

**Figure 5:**
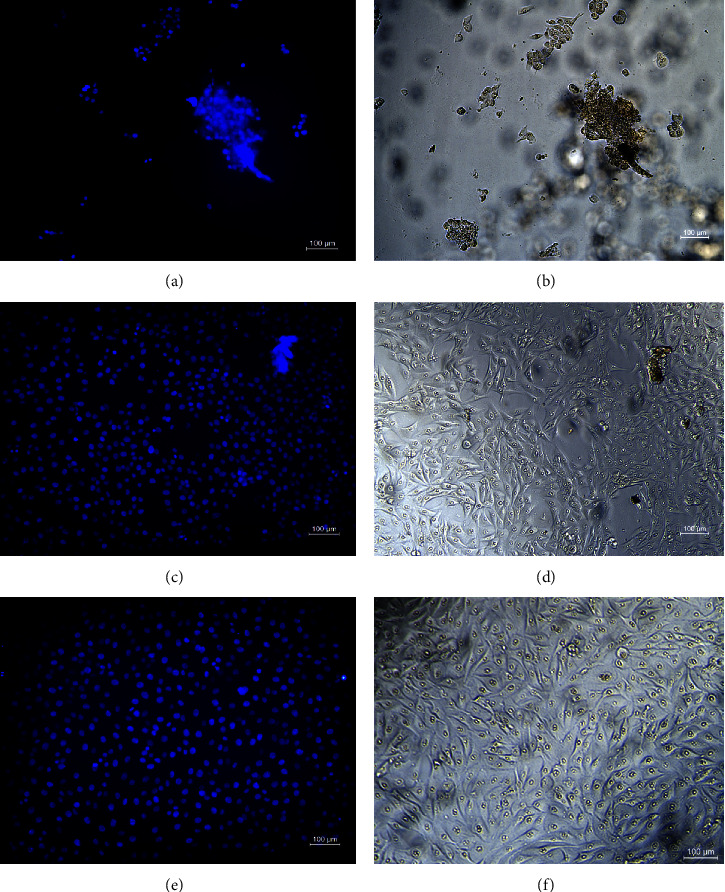
Cell adhesion surface test using the MG63 cell line and *Eupsophus vertebralis* secretions assays to: (a, b) EVPN1370 died samples (c, d) EVPN1040 dried samples, and (e, f) controls. Fluorescence of the nuclei labelled with DAPI (left) and light microscopy (right) is shown. Scale bar = 100 *μ*m.

In the same way, we propose to study the cytotoxicity of the secretions to understand the effect of the samples and their minor compounds on cell viability (Figure 4). The effect of the secretions on cells was tested by indirect exposure, in which the cells were exposed to the extract (Figure 4(a)), and direct contact, in which the cells were seeded on the surface of the hydrogel coating (Figure 4(b)). Surprisingly, one of the secretions (EVPN1370) exhibited a strong cytotoxic profile, while the other secretions (EVPN1040, EVMO1417, and EVMO1418) did not demonstrate any cytotoxic effects (Figure 4). Instead, they served as effective supports for cell attachment and proliferation when tested in direct contact with the cells (Figure 5 and Supplementary Figure 1). One possible explanation for the cytotoxic effect of EVPN1370 secretion is the presence of small, potent molecules that are weakly bound to the proteins that form the hydrogel. When inserted in the culture medium for extraction, these molecules could be released and exert their toxic effect, killing the cells. This hydrogel with such cytotoxic potential could be deeply studied, aiming to look for the cytotoxic molecules and to isolate them to evaluate their antitumor effect. With respect to the secretions EVPN1040, EVMO1417, and EVMO1418, their noncytotoxic effect and the attachment of cells on their surface indicate their potential in applications such as scaffolds/supports for cell growth for use in regenerative medicine, as well as matrices for application in drug delivery, among other possibilities. Interestingly, EVPN1040, one of these noncytotoxic samples, could form films when dried (Figure 1(b)), which could widen its range of applications for use as a wound healing material.

All secretions analyzed exhibited similar chemical structures, as demonstrated by the FTIR results (Figure 2), and displayed comparable rheological properties in the wet state (Figure 3), forming robust hydrogels. In the dry state, EVPN1370, EVMO1417, and EVMO1418 formed very rigid grain aggregates (Figure 1(c)), and EVPN1040 formed a transparent and visually homogeneous film (Figure 1(b)). Although secretions from four samples did not exhibit antimicrobial effects (Supplementary Figure 1), their influence on cell growth varied. In fact, EVPN1370 displayed a high toxicity, while the other samples were nontoxic (Figures 4 and 5 and Supplementary Figure 2). The nontoxic secretions, especially from Punucapa, are therefore promising candidates for future investigations in the field of medical hydrogels. Another important property of these secretions is their adhesive properties, as previously shown in our report [19]. Although the samples were collected from the same animal species, potential limitations of this study could be related to the conditions at the time of sample collection, which might influence the composition of the secretions. It is possible that the sampling location, gender, and emotional condition led to the release of defense substances into the secretions, and these substances displayed toxic effects on the cells. Although these results can be seen as the first findings with respect to the activity of these secretions, this opens the opportunity for widening the studies in several fields of science, including the materials development and biological science.

## 4. Conclusions

The search for new natural biomaterials for healing tissues, adhesives, drug delivery, and substances with biocide properties, such as antimicrobial and antitumoral activity, is growing rapidly. The demand for new sources of biomaterials is high, aiming to reveal their properties and encourage advances in medical science. Here, we have described the rheological, antimicrobial, and cytotoxic activity of the secretion of individuals from *Eupsophus vertebralis* species, as the preliminary study and the first level of evidence for future biomedical applications of these secretions. Our results showed the robustness of the secretions as hydrogels and indicated that the crude biomaterial can be used as a source for the extraction of potent eukaryotic biocidal molecules. In addition, the cytocompatible hydrogels are promising materials for use as hydrogels for drug and cell delivery, and for the development of new biological adhesives.

## Data Availability

The data used to support the findings of this study are included within the article and the supplementary information file.

## Supplementary Materials

Supplementary Materials Supplementary Figure 1: plate diffusion assay to test antimicrobial effect using secretions from EVPN1370 and EVPN1040 against *S. aureus* (ATCC25923) and *E. coli* (ATCC25922). Supplementary Figure 2: cell adhesion surface test using the MG63 cell line and *Eupsophus vertebralis* secretions. Assays correspond to (a, b) EVMO1417 dried samples, (c, d) EVMO1418 dried samples and (e, f) controls. Fluorescence of the nuclei labeled with DAPI (left) and light microscopy (right) are shown.

## References

[1] Khan R. A. (2018). Natural products chemistry: the emerging trends and prospective goals. *Saudi Pharmaceutical Journal*.

[2] Clarke B. T. (1997). The natural history of amphibian skin secretions, their normal functioning and potential medical applications. *Biological Reviews of the Cambridge Philosophical Society*.

[3] Cseke L. J., Kirakosyan A., Kaufman P. B., Warber S., Duke J. A., Brielmann H. L. (2016). *Natural Products from Plants*.

[4] Jabeen S., Hanifa M. A., Khanb M. M., Qadric R. W. K. (2014). Natural products sources and their active compounds on disease prevention: a review. *International Journal of Chemical and Biochemical Sciences*.

[5] Amri E., Mamboya F. (2012). Papain, a plant enzyme of biological importance: a review. *American Journal of Biochemistry and Biotechnology*.

[6] Cooper R., Nicola G. (2014). *Natural Products Chemistry: Sources, Separations and Structures*.

[7] Fan Q., Ma J., Xu Q. (2015). Animal-derived natural products review: focus on novel modifications and applications. *Colloids and Surfaces B: Biointerfaces*.

[8] Lee K. H. (2010). Discovery and development of natural product-derived chemotherapeutic agents based on a medicinal chemistry approach. *Journal of Natural Products*.

[9] Salehi B., Sestito S., Rapposelli S. (2018). Epibatidine: a promising natural alkaloid in health. *Biomolecules*.

[10] Spande T. F., Garraffo H. M., Edwards M. W., Yeh H. J. C., Pannell L., Daly J. W. (1992). Epibatidine: a novel (chloropyridyl)azabicycloheptane with potent analgesic activity from an Ecuadoran poison frog. *Journal of the American Chemical Society*.

[11] Rodriguez C., Rollins-Smith L., Ibanez R., Durant-Archibold A. A., Gutierrez M. (2017). Toxins and pharmacologically active compounds from species of the family Bufonidae (Amphibia, Anura). *Journal of Ethnopharmacology*.

[12] Brizzi R. D. G., Jantra S., Jamieson B. G. M. (2003). An overview of breeding glands. *Reproductive Biology and Phylogeny of Anura: Cap. 6*.

[13] Evans C. M., Brodie E. D. (1994). Adhesive strength of amphibian skin secretions. *Journal of Herpetology*.

[14] Phillips B., Shine R. (2007). When dinner is dangerous: toxic frogs elicit species-specific responses from a generalist snake predator. *The American Naturalist*.

[15] Böker K. O., Richter K., Jäckle K. (2019). Current state of bone adhesives—necessities and hurdles. *Materials*.

[16] Channing A. E. (2001). *Amphibians of Central and Southern Africa*.

[17] Tyler M. J., von Byern J., Grunwald I. (2010). Adhesive dermal secretions of the amphibia, with particular reference to the Australian Limnodynastid genus *Notaden*. *Biological Adhesive Systems from Nature to Technical and Medical Application*.

[18] Graham L. D., Glattauer V., Peng Y., Smith A. M. (2016). An adhesive secreted by Australian frogs of the genus *Notaden*. *Biological Adhesives*.

[19] Suárez-Villota E. Y., Trovatti E., Contreras F. A., Nuñez J. J. (2021). Characterisation of a skin secretion with adhesive properties in the ground frog *Eupsophus vertebralis* (Alsodidae). *Herpetozoa*.

[20] Blotto B. L., Nuñez J. J., Basso N. G., Ubeda C. A., Wheeler W. C., Faivovich J. (2013). Phylogenetic relationships of a Patagonian frog radiation, the *Alsodes* + *Eupsophus* clade (Anura: alsodidae), with comments on the supposed paraphyly of *Eupsophus*. *Cladistics*.

[21] Lu X., Che Q., Lv Y. (2010). A novel defensin-like peptide from salivary glands of the hard tick, Haemaphysalis longicornis. *Protein Science*.

[22] Page B., Page M., Noel C. (1993). A new fluorometric assay for cytotoxicity measurements *in-vitro*. *International Journal of Oncology*.

[23] Chen Q., Liang S., Thouas G. A. (2013). Elastomeric biomaterials for tissue engineering. *Progress in Polymer Science*.

[24] Quagliata S., Malentacchi C., Giachi F., Delfino G. (2008). Chemical skin defence in the Eastern fire-bellied toad *Bombina orientalis*: an ultrastructural approach to the mechanism of poison gland rehabilitation after discharge. *Acta Herpetologica*.

[25] Delfino G., Brizzi R., Melis G. (1996). Merocrine secretion from serous cutaneous glands in *Rana esculenta* complex and *Rana iberica*. *Alytes*.

[26] Holmes C. H., Moondi P. S., Rao R. R., Balls M. (1977). *In vitro* studies on the effects on granular gland secretion in *Xenopus laevis* skin of stimulation and blockade of alpha and beta adrenoceptors of myoepithelial cells. *Cell Biology International Reports*.

[27] von Byern J., Grunwald I., Kosok M. (2017). Chemical characterization of the adhesive secretions of the salamander *Plethodon shermani* (Caudata, Plethodontidae). *Scientific Reports*.

[28] Barth A. (2007). Infrared spectroscopy of proteins. *Biochimica et Biophysica Acta (BBA)- Bioenergetics*.

[29] Wu R., McMahon T. B. (2009). Protonation sites and conformations of peptides of glycine (Gly1−5H+) by IRMPD spectroscopy. *The Journal of Physical Chemistry B*.

[30] Bashir S., Hina M., Iqbal J. (2020). Fundamental concepts of hydrogels: synthesis, properties, and their applications. *Polymers*.

[31] Parhi R. (2017). Cross-linked hydrogel for pharmaceutical applications: a review. *Advanced Pharmaceutical Bulletin*.

[32] Whittaker J., Balu R., Choudhury N. R., Dutta N. K. (2014). Biomimetic protein-based elastomeric hydrogels for biomedical applications. *Polymer International*.

[33] Ladram A., Nicolas P. (2016). Antimicrobial peptides from frog skin: biodiversity and therapeutic promises. *Frontiers in Bioscience*.

