# Supplementary material for: Rheological and Biological Properties of Adhesive Skin Secretions from Eupsophus vertebralis (Anura: Alsodidae)
Source: Scientifica (Cairo). 2024 Mar 25;2024:2722351. doi: 10.1155/2024/2722351 (PMC10985274; doi:10.1155/2024/2722351)
Supplement: Supplementary Materials — Supplementary Figure 1: plate diffusion assay to test antimicrobial effect using secretions from EVPN1370 and EVPN1040 against S. aureus (ATCC25923) and E. coli (ATCC25922). Supplementary Figure 2: cell adhesion surface test using the MG63 cell line and Eupsophus vertebralis secretions. Assays correspond to (a, b) EVMO1417 dried samples, (c, d) EVMO1418 dried samples and (e, f) controls. Fluorescence of the nuclei labeled with DAPI (left) and light microscopy (right) are shown. [file 2722351.f1.docx]

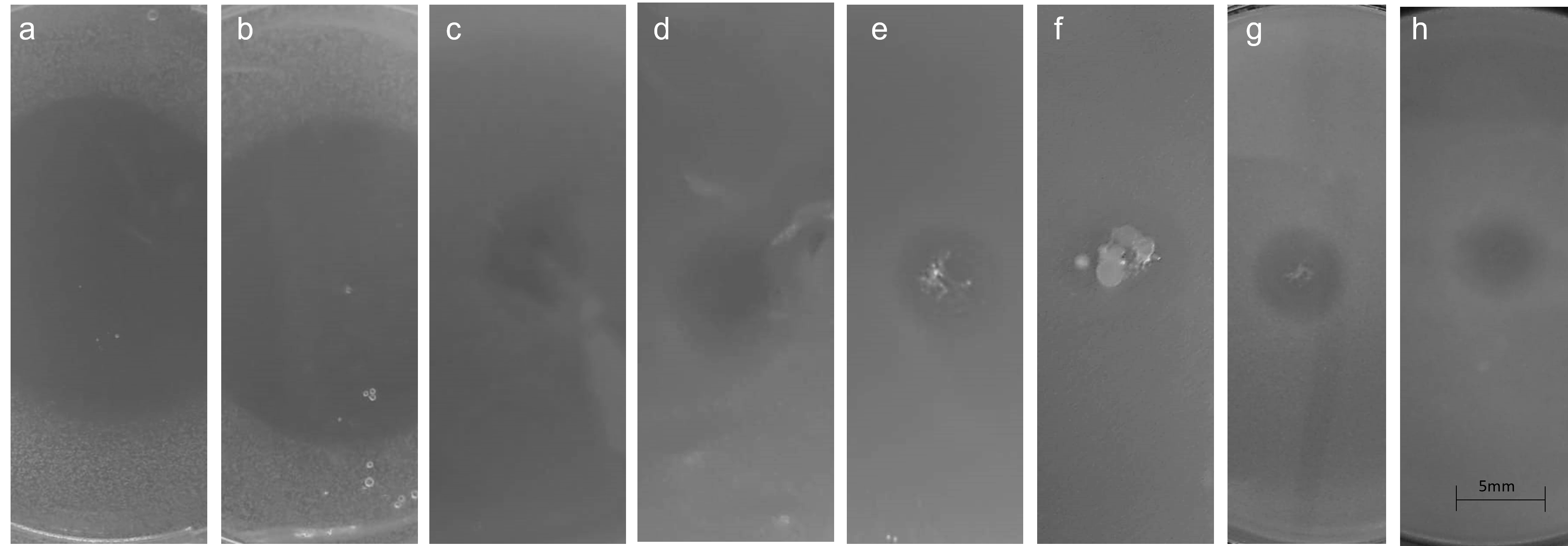


**Supplementary Figure 1.** The Eupsophus vertebralis secretions (EVPN1370 -EVPN1040) subjected to an antimicrobial effect test using a plate diffusion assay. a. Positive control (Antibiotic-Antimycotic) against S. aureus (ATCC 25923). b. Positive control (Antibiotic-Antimycotic) against E. coli (ATCC 25922). c. Negative control (Acetic Acid) against S. aureus (ATCC 25923). d. Negative control (Acetic Acid) against E. coli (ATCC 25922). e. EVPN1370 against S. aureus (ATCC 25923) f. EVPN1370 against E. coli (ATCC 25922). g. EVPN1040 against S. aureus (ATCC 25923). h. EVPN1040 against E. coli (ATCC 25922). Note no detectable antimicrobial effect from the raw secretion, as the inhibition radius was equivalent or smaller than the negative control (6 mm)


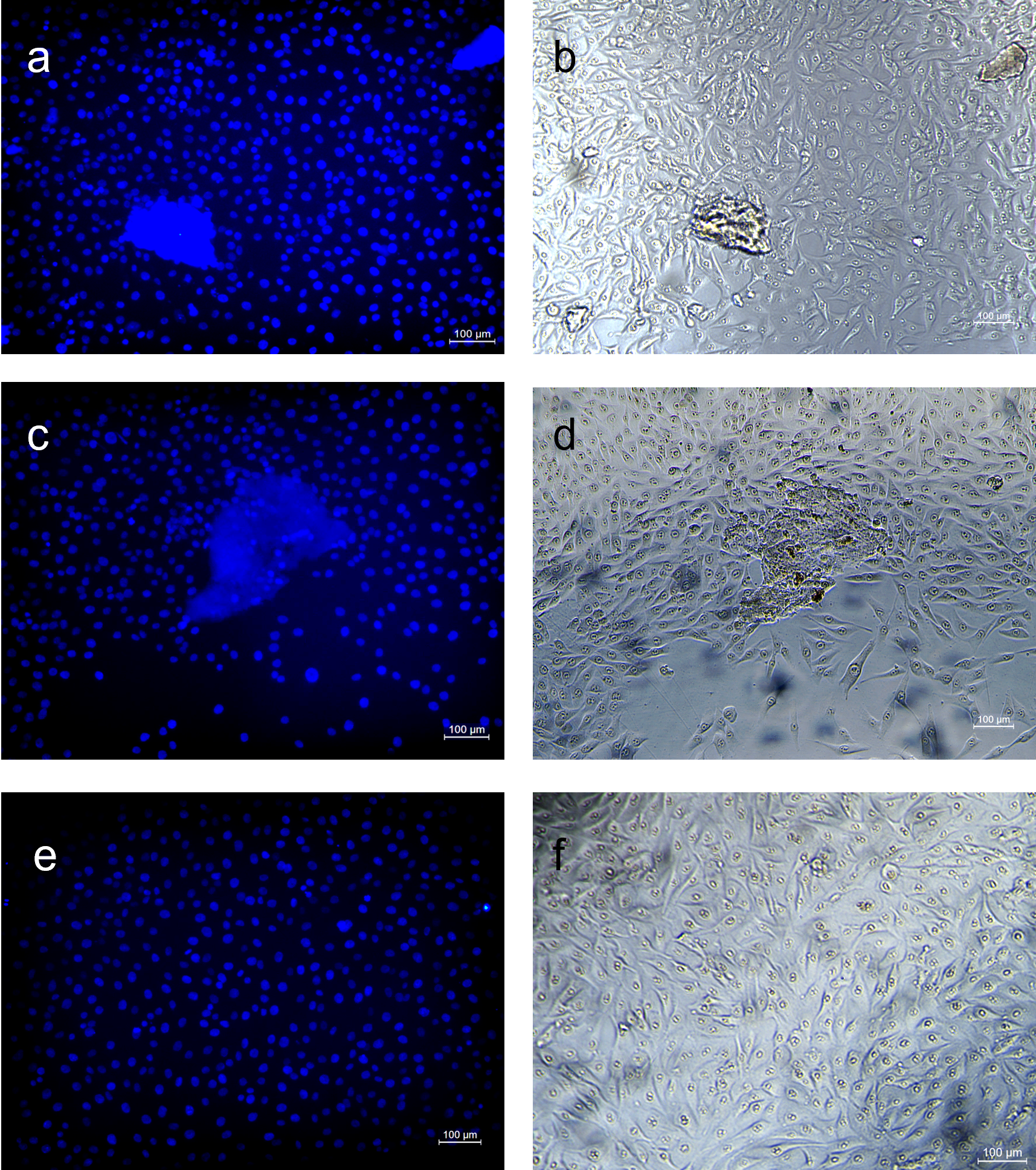


**Supplementary Figure 2.** Cell adhesion surface test using the MG63 cell line and Eupsophus vertebralis (format: italic) secretions. Assays correspond to (a, b) EVMO1417 dried samples, (c, d) EVMO1418 dried samples and (e, f) controls. Fluorescence of the nuclei labeled with DAPI (left) and light microscopy (right) are shown.
